# Now Is the Time to Strengthen Government-Academic Data Infrastructures to Jump-Start Future Public Health Crisis Response

**DOI:** 10.2196/51880

**Published:** 2024-04-24

**Authors:** Jian-Sin Lee, Allison R B Tyler, Tiffany Christine Veinot, Elizabeth Yakel

**Affiliations:** 1 School of Information University of Michigan Ann Arbor, MI United States; 2 UK Data Archive University of Essex Colchester United Kingdom; 3 Department of Health Behavior and Health Education School of Public Health University of Michigan Ann Arbor, MI United States; 4 Department of Learning Health Sciences School of Medicine University of Michigan Ann Arbor, MI United States

**Keywords:** COVID-19, crisis response, cross-sector collaboration, data infrastructures, data science, data sharing, pandemic, public health

## Abstract

During public health crises, the significance of rapid data sharing cannot be overstated. In attempts to accelerate COVID-19 pandemic responses, discussions within society and scholarly research have focused on data sharing among health care providers, across government departments at different levels, and on an international scale. A lesser-addressed yet equally important approach to sharing data during the COVID-19 pandemic and other crises involves cross-sector collaboration between government entities and academic researchers. Specifically, this refers to dedicated projects in which a government entity shares public health data with an academic research team for data analysis to receive data insights to inform policy. In this viewpoint, we identify and outline documented data sharing challenges in the context of COVID-19 and other public health crises, as well as broader crisis scenarios encompassing natural disasters and humanitarian emergencies. We then argue that government-academic data collaborations have the potential to alleviate these challenges, which should place them at the forefront of future research attention. In particular, for researchers, data collaborations with government entities should be considered part of the social infrastructure that bolsters their research efforts toward public health crisis response. Looking ahead, we propose a shift from ad hoc, intermittent collaborations to cultivating robust and enduring partnerships. Thus, we need to move beyond viewing government-academic data interactions as 1-time sharing events. Additionally, given the scarcity of scholarly exploration in this domain, we advocate for further investigation into the real-world practices and experiences related to sharing data from government sources with researchers during public health crises.

## Introduction

Although the world appears to be recovering from the intense impact of the COVID-19 pandemic, it is imperative to recognize that the far-reaching effects of this disease continue to endure across the globe. Its significant ramifications, such as economic fallout, disruptions in education, mental health challenges, and racial and socioeconomic health inequities, have left indelible marks on humanity, showcasing the vulnerabilities of human society when confronted with a global public health crisis. Looking back, we should now ask: “What actions could have been done better to save more lives and reduce the aforementioned negative effects?” Moving forward, it is crucial to reflect on the valuable lessons that appear when we pose this question. Such reflection may prepare us better for future public health crises.

Amid any effort to effectively manage the spread of the COVID-19 pandemic, the rapid sharing of data stands out as a key strategy. However, instances of failure have been exposed, signaling opportunities for improvement in this critical facet of the pandemic response. Just 1 year into the COVID-19 outbreak, the British Medical Association’s chair of council voiced criticism, suggesting that “our devastating mortality figures could in part be a result of the failure of the government to properly and openly share data, communicate accurately, and act swiftly” [1]. He stressed the problematic absence of transparency concerning the actual availability of personal protective equipment supplies and the formulation of decisions on restrictions and tiers (ie, a classification system indicating local COVID-19 alert levels adopted in the United Kingdom) without also revealing the underlying statistics that supported them [1]. The US Centers for Disease Control and Prevention (CDC) itself faced criticism for its perceived “slow and siloed approach to sharing data,” which resulted in overly optimistic evaluations of the evolving vaccine effectiveness against the delta variant and contributed to the country’s falling behind in addressing that new viral mutation [2].

Government entities, a primary source of public health data, bear the responsibility for ensuring timely availability and facilitating the subsequent use of such data. To expedite the pandemic response efforts, social debates and academic studies have centered on enhancing data sharing among health care providers [3], across government departments at different levels [4], and on an international scale [5]. However, a data sharing approach that receives less attention but carries equal significance during public health crises is cross-sector data sharing collaborations between government entities and academic researchers. Specifically, this involves dedicated projects wherein a government entity shares the public health data it aggregates with an academic research team for further data analysis. The resulting data insights are then leveraged to inform policy making.

In this viewpoint, we outline the common challenges in data sharing during COVID-19, as well as other public health and general crises such as natural disasters and humanitarian emergencies. We then argue that government-academic data collaboration has the potential to alleviate these challenges, making it a topic worthy of deeper scholarly exploration. We aim to initiate a constructive discussion on effective strategies to foster this kind of cross-sector collaboration, thus paving the way for more robust and resilient responses to future public health crises.

## The Significance of Data Sharing During Crises Cannot Be Overstated

In times of crisis, the saying “speed is everything” resonates more profoundly than ever. The landscape of a crisis is defined by difficulties related to uncertainty, urgency, and the relentless pursuit of solutions. Among these difficulties, data emerge as a beacon of understanding—a key resource that can illuminate facts, accelerate actions, and prevent unnecessary public panic. The significance of data sharing cannot be overstated during such moments when rapid access to comprehensive, accurate, and timely data becomes the linchpin of effective crisis management.

The lessons learned from previous natural disasters and humanitarian crises have reinforced the importance of rapid data sharing [6-8]. This demand originated from not only frontline responders, decision makers, and those directly impacted but also those capable of and willing to contribute to crisis resolution, such as academic researchers. Similarly, even before the arrival of the unprecedented COVID-19 pandemic, a collection of public health crises highlighted the critical role of sharing data to safeguard global well-being. The outcomes of not sharing data or failing to integrate and coordinate data access during public health emergencies were studied, as seen during the 2003 outbreak of SARS [9,10]. In the past, data sharing was limited by technological constraints or underdeveloped legal frameworks. However, the necessity of data exchange was evident. Today, with advancements in data science knowledge and techniques, data sharing holds the potential for even greater advances that may improve the responses to public health crises.

Several characteristics collectively set COVID-19 apart from preceding crisis events: its expansive geographical reach, rapid transmission, prolonged duration, and far-reaching socioeconomic consequences. Moreover, the exceptional need for rapid data dissemination across jurisdictional and organizational boundaries during this crisis also added to its distinctiveness [11]. As stated by epidemiologist Dr Maria van Kerkhove, the COVID-19 technical lead at the World Health Organization (WHO), “When there’s so little information on a novel pathogen, any information that you can get your hands on is absolutely critical” [12]. During the COVID-19 pandemic, data sharing has played a crucial role in enhancing decision makers’ contextual understanding of the pandemic, which was deemed a key aspect of “public health situational awareness” by the US government during the pandemic [13]. Ideally, such heightened awareness would have facilitated more rapid responses, thus enabling the implementation of appropriate measures, such as resource allocation and public health interventions. When successful, data sharing also enables the examination of health disparities by providing access to demographic data, which was particularly significant in COVID-19 due to the disproportionate impact on marginalized socioeconomic, racial, and ethnic groups worldwide [14,15].

During the early stages of COVID-19, we did witness efforts to share data, such as information about potential treatments and disease spread. However, in many instances, the underlying data were of varying or low quality, as evidenced by the pandemic-related preprints that emerged [16]. In addition, considering the unpredictability of the COVID-19 pandemic, rapid data exchange necessitated effective coordination particularly among various stakeholders engaged in the “learning health system” cycle [17], which involved assembling, analyzing, and transforming data into knowledge and then performance, all within a limited time frame. Different actors’ data needs also varied across pandemic phases, such as whether a situation occurred suddenly or insidiously or whether consequences rapidly abated or lingered. Given the complexity of crisis data–sharing relationships, in the next section, we examine documented challenges in data sharing during crises that provide us with a better understanding of this issue.

## Data Sharing Challenges During Public Health Crises

Public health crises have the potential for global ramifications; however, addressing them necessitates a localized approach [18]. During the COVID-19 outbreak, there existed attempts to share key data points (eg, viral genomes and methods of transmission) at the international, national, and local levels [19]. However, a difficulty lay in effectively acquiring public health insights due to the complexity of integrating disparate data across geopolitical boundaries and jurisdictional levels.

In the United States, the COVID-19 response was impaired due to the public health data infrastructures’ inability to effectively share data across and within jurisdictions. As widely reported, the efficiency and timeliness of data sharing, supported by these data infrastructures, have not always been satisfactory. The desired data are “scattered” across unconnected or proprietary databases, exist in incompatible formats, or are of dubious quality and provenance [20]. Present methods of collecting public health data primarily depend on manual processes for reporting instances of certain communicable diseases and outbreaks of new diseases. Using the data accessible in electronic health record systems is not often done, even when possible. This disconnect impedes the effective use of available data. Unsurprisingly, secondary use of clinical data for public health purposes is usually insufficient [21]. Furthermore, many researchers were and still are unable to swiftly integrate with existing public health data infrastructures and “find the right antidote” for research demands when facing unforeseen public health crises [22].

The long-standing underinvestment in the maturity and agility of US public health data infrastructures has been frequently emphasized at the federal as well as state and local levels [23]. This gap became particularly problematic during the COVID-19 pandemic. From a broader perspective, neither early legislative efforts, such as the HITECH Act of 2009, nor more recent programs dedicated to COVID-19, such as the CDC’s Data Modernization Initiative, have successfully addressed the country’s fragilities in constructing critical infrastructures to meet the ongoing public health surveillance needs [21]. Admittedly, modern data-driven technologies have been widely implemented in both research and clinical contexts. Nevertheless, the aforementioned deficiencies hindered not only comprehensive real-time data analyses at the technical level [24] but also large-scale coordination between data holders and requesters at the social and organizational levels [25]. For example, in response to COVID-19, a plethora of data aggregation initiatives emerged, involving key actors, such as academic medical center networks as data holders and public health departments as data consumers. Subsequently, data requests from these federal- or state-level public health departments turned out to impose a substantial burden on academic medical centers’ reporting mechanisms [25].

While measures taken in response to the COVID-19 pandemic brought some data sharing challenges into sharp relief, the challenges arising at that time have a long history. A succession of diverse crises exposed the challenges that responders encountered when aiming to achieve rapid data sharing. By mapping the nature of different types of crises alongside their documented challenges, we can enhance our understanding of the most important data sharing approaches to study and maintain during such crises. In Table 1 [25-97], based on the extant research literature, we summarize 5 categories and 20 subcategories of common data sharing challenges during COVID-19, other public health crises, as well as other natural disasters and humanitarian crises.

After conducting a comprehensive search of scholarly literature in various fields such as public health, biomedicine, crisis informatics, and broader information science, we developed a corpus of papers relevant to data sharing challenges. Starting from this corpus, the aforementioned categories and subcategories were developed through an iterative qualitative analysis process to describe, conceptually order, and classify the bodies of text [98] as follows. First, we discerned challenges documented in the literature. Identifiable patterns then emerged, leading to the inductive grouping of specific challenges into common broad categories. These categories were further refined into their respective subcategories, taking into account the nuances of specific challenges and whether they were experienced during the COVID-19 pandemic, other public health crises, or additional types of crises (ie, natural disasters and humanitarian crises). Given the vast volume of publications discussing data sharing challenges, the categories and subcategories we developed are not meant to be exhaustive. Rather, they function as a starting framework for an interpretive critique of existing research gaps.

In all, the 5 categories of data sharing challenges we have identified are (1) data availability and quality, (2) data management and sharing, (3) information systems and data interoperability, (4) resource limitations, and (5) multiparty collaboration and coordination. In Table 1, we use omnibus terms, such as “stakeholders” and “data sharing entities,” to encompass a diverse range of individuals (eg, researchers, clinicians, and first responders) and organizations (eg, government agencies, health care providers, and humanitarian groups) actively involved in data exchange during crises. In the next section, we describe government-academic data collaborations—an often-underexplored type of data interaction during public health crises—and how to mitigate the challenges outlined below in these collaborations.

**Table 1:** Common data sharing challenges among stakeholders and example references by type of crises.

Categories and subcategories			Definitions		Example references				
					COVID-19		Other public health crises		Natural disasters and humanitarian crises
**Category 1: data availability and quality**									
	Data collection for dynamic needs	Data needs can vary across different crisis phases and communities. Collecting data within a limited time frame and managing duplicated data requests from multiple parties could overly burden a data sharing entity. Solely gathering minimal data may also render them unsuitable for comprehensive use.		[25-28]		[29-31]		[6,32,33]	
	Data location uncertainties and awareness gaps	Stakeholders are generally unaware of the availability or whereabouts of potentially useful data and associated technologies.		[34-36]		[31,37-39]		[7,33,40]	
	Information overload	An overwhelming amount of information or data is accessible, yet organizations in need lack the sufficient capacity to effectively absorb and identify the most pertinent data points that they require.		[12,41-43]		[10,44]		[45-47]	
	Questionable data quality	Due to the time constraints for data collection, data quality may suffer, leading to doubts from data users or the general public regarding the credibility of data sharing entities. These doubts, in turn, raise concerns about the potential for misguided decisions based on the data.		[12,43,48,49]		[38,50,51]		[47,52,53]	
**Category 2: data management and sharing**									
	Poor or inconsistent data management practices	Stakeholders exhibit inadequate or divergent data management practices (eg, little documentation of provenance, version control, data dictionaries, or workflows and a lack of standardized procedures). These issues are further intensified by the urgency of crisis response efforts. Ensuring the long-term management of shared data is another notable challenge.		[26,34,54]		[55-57]		[58-60]	
	Insufficient data sharing frameworks	Insufficient data sharing policies or agreements are in place either within or between organizations to facilitate both the exchange of data and the willingness of stakeholders to participate in such activities.		[61-63]		[50,64,65]		[28,59,66]	
	Perceived risks for sharing data	Data holders may recognize the drawbacks that data sharing can pose to their self-interest (eg, the economic losses or governance issues that a nation may experience after reporting pandemic information internationally) and become hesitant to engage in such activities.		[41,67,68]		[37,56,69]		[6,53,70]	
	Tensions between openness and privacy	Concerns arise among stakeholders and the general public regarding the imperative to uphold data subject privacy and strike a balance between privacy protection and the timely sharing of data.		[71-73]		[57,65,74]		[40,53,75]	
**Category 3: information systems and data interoperability**									
	Fragmented data landscape	The data of interest are dispersed across numerous unconnected or proprietary databases, often in incompatible formats.		[24,34,71]		[39,55,76]		[60,66,77]	
	Antiquated or aging information systems and unsuccessful adoption of technologies	The existing IT systems holding the data may be outdated, resulting in manual operations for data sharing and related activities. In certain cases, under-resourced crisis sites lack well-implemented technological solutions to enable timely data sharing.		[72,78,79]		[44,76,80]		[32,66,75]	
	Data exchange and processing at scale	Data pipelines and infrastructures are largely absent or unprepared to exchange, manage, and analyze the growing volumes of data for crisis response.		[24,27,48]		[38,81]		[47,82,83]	
	Data standardization barriers	The absence of interoperable transactional- and data-level standards hinders the use of existing data tools and systems between organizations.		[19,26,84,85]		[50,76,86]		[33,45,59]	
**Category 4: resource limitations**									
	Deficiency of data-related workforce	There is often a lack of data-related workforce or capacities embedded in crisis response teams.		[5,62,80]		[30,44,87]		[52,88-90]	
	Absence of incentive structures	Data holders often exhibit reluctance to share data that they have expended significant efforts to collect and curate. By sharing data, others may make discoveries before the data holders do and gain a competitive advantage.		[19,36,64]		[9,38,86,91]		[6,88,92]	
	Inadequate legal support	Existing legal frameworks regarding privacy and others that regulate data sharing are either inadequate or overly intricate and may not be optimally suited for swiftly evolving crises.		[5,61,79]		[51,74,93]		[82,89,92]	
	Difficulties in reallocating resources for crisis response	Financial and human resources are often directed to specific areas or for specific needs, or cannot easily or at will be allocated to specific tasks.		[35,71,78,94]		[10,31,55,87]		[32,40,58]	
**Category 5: multiparty collaboration and coordination**									
	Extensive geographic coverage and intricate geopolitics	There is a lack of shared data tools across jurisdictions (eg, a metropolitan area spanning multiple states) for pandemic control activities, such as case management, contact tracing, and disease modeling.		[62,63,84]		[29,30,93]		[70,90]	
	Missing coordination mechanisms	There is a lack of coordinating bodies or channels to help manage data sharing requests across multiple entities that hold or seek access to data.		[25,35,61,85]		[10,29,54]		[7,95,96]	
	Misaligned goals or competitive priorities	Divergent goals and priorities in crisis response efforts among organizations can lead to their eventual absence or diminished participation in data sharing activities.		[27,62]		[29,39,93]		[45,82,92]	
	Organizational politics, bureaucracy, and power dynamics	Due to prevailing power structures or organizational norms, data sharing entities may exhibit reluctance to provide data to avoid interference from hierarchically superior or external entities.		[67,84,94]		[37,69,97]		[8,75,95]	

## Navigating Challenges: Government-Academic Collaboration as Part of the Social Infrastructure for Public Health Crisis Response

Data collected, aggregated, or provided by government entities have been observed to play a key part in managing the COVID-19 crisis. As an example that involves government data for internal use, in the United States, the city government of Boston used its preexisting data warehouse, aggregating data from 31 departments, to rapidly develop a public dashboard at the outset of the pandemic [99].

Simultaneously, there were instances globally where academia has engaged in using government data to understand the pandemic, with various forms of such cross-sector initiatives. For example, the Israeli government orchestrated a “datathon” competition—an event uniting participants from diverse sectors, including academic scientists, to devise practical, data-driven models and insights [100]. Another approach to effectively leveraging government data is by directly making them available to citizens, including academic researchers. The term “open government data” (OGD) refers to government-held data made accessible to the public to enhance transparency regarding government operations [101]. One common method for obtaining OGD is through government agencies’ open data portals [102]. This method has enabled various stakeholders to engage in data analysis before and during the COVID-19 period [103,104].

Nonetheless, there are limitations to the aforementioned data interactions between government entities and academia. Specifically, short-term data analysis competitions may not adequately support the relatively medium to long-term needs for pandemic response policy planning by government entities. Participants from various sectors are unlikely to sustainably remain within the government's data collaboration network after the competition ends. On the other hand, in relation to OGD, there is a critical aspect of their availability—when accessing OGD, users download data independently without direct interactions with government agencies as data providers. Such a relatively unilateral data access approach may give rise to issues related to data quality and usability (eg, data integrity, granularity, and timeliness [105]), potentially impeding users’ understanding and appropriate interpretation of OGD and in turn undermining the data’s overall effectiveness to be used accurately, appropriately, and efficiently.

Consequently, there is a growing demand for enhanced “direct” collaboration between government entities and researchers on data-centric research projects. This form of collaboration entails government entities sharing data with researchers who have advanced data science skills, leading to productive “research-policy partnerships” that subsequently inform policy-making processes [106]. When successful, such partnerships serve as a catalyst for “rapid response data science” to address public health crises [107]. Particularly, governments possess valuable data but may lack the necessary resources to analyze them [108], while academic researchers often face challenges in collecting or accessing critical data due to legal, technical, or financial limitations [109]. Consequently, an ideal scenario involves researchers with data science and other methodological expertise effectively using these public health data to conduct studies that surpass the capabilities of the government’s in-house efforts [110], thereby facilitating greater evidence-based policymaking. Notably, beyond merely handing over the data, governmental collaborators play a role in identifying the critical problems to be solved, as well as in pinpointing the strengths and limitations of the shared data. This helps academic collaborators develop solutions that truly address the issues at hand and mitigate the risk of misinterpretation or misuse of the data they acquire. However, despite the needs and benefits, there is a lack of in-depth investigations into the precise nature of such cross-sector collaborations driven by data flows specifically from government entities to academic researchers.

Transitioning into the postpandemic era, the current juncture presents an opportune moment for conducting more systematic research into the aforementioned form of government-academic data collaborations, whether examining individual cases or identifying patterns across multiple cases. Notably, the sole, relatively detailed examination of similar government-researcher data collaborations during COVID-19 that we are aware of is a report [111] revealing the partnership between the Washington State Department of Health (DOH), the University of Washington, and multiple research institutes, including the Institute for Disease Modeling (IDM), which operates as an embedded research group in a nonprofit foundation. Within this collaboration, the Washington State DOH was able to successfully share data with IDM, ultimately leveraging insights derived from IDM researchers’ modeling and analytical findings to inform the state’s pandemic response strategies.

We possess limited information about the development and maintenance of other government-academic collaborations, primarily in the United States. These include a project in which a Stanford University team of data modeling experts used data provided by California state agencies to forecast disease trends for public health officials [112]. Also in California, in response to the temporary closure of most daycare centers, the state’s Health and Human Services Agency and the University of Southern California built on their preexisting data integration program, attempting to connect essential workers with available childcare providers [113]. Additionally, a multidisciplinary team at the University of Michigan partnered with the Michigan Department of Health and Human Services to develop a series of data-driven tools (eg, symptom and vaccination monitoring applications) to track the pandemic within the state [114]. Despite this limited information, these additional cases make it clear that such collaborations exist more broadly, and that they potentially hold value in responding to public health crises.

Our call for research into government-academic collaborations is vital given the history of previous efforts. For example, between 2005 and 2011, the CDC launched the Centers of Excellence in Public Health Informatics program. This initiative shared similarities with the form of government-academic collaboration we are advocating as it aimed to bridge the gap between public health research and practice through collaborations among academia, local or state public health departments, and other health informatics professionals [115]. The program financially supported academic institutions, such as Harvard University and Indiana University [116], to establish research centers that would translate research outcomes into public health practice. Data sharing and information exchange were integral components as well, though primarily from clinical sources to public health information systems [117]. However, after the funding concluded, the infrastructures of the research centers necessitated institutional or external backing for sustainability. This indicates the difficulty in maintaining the long-term viability of such short- or medium-term programs.

Up to this point, we have outlined several forms of government-academic data interactions and their limitations in terms of effectiveness, efficiency, and sustainability. In the following subsection, we elaborate on an alternative collaborative form that may function better during public health crises than the other aforementioned government-academic data interactions.

## Government-Academic Collaborations as a Promising Solution to Data Sharing Challenges

### Overview

A public health data infrastructure can be defined as “an ecosystem composed of the people, processes, procedures, tools, facilities, and technologies, which supports the capture, storage, management, exchange, and creation of data and information to support individual patient care and population health” [118]. Nonetheless, it is notable that existing discussions around these kinds of infrastructures mostly center on technological aspects, such as health information systems and their data standards for interoperability. At the same time, other key components involving people, processes, and norms have received less attention [119]. In fact, for researchers, we contend that data collaborations with government entities should be considered part of the social infrastructure that supports their research efforts toward public health crisis response. Specifically, as we argue below, many of the data sharing challenges displayed in Table 1 may be effectively mitigated through well-planned government-academic collaborations. In the upcoming sections, we explain how the 5 types of challenges (data availability and quality, data management and sharing, information systems and data interoperability, resource limitation, and multiparty collaboration and coordination) can be navigated by developing partnerships between government entities and academia, particularly during public health emergencies:

### Data Availability and Quality

Government-academic data collaborations are goal oriented and usually provide researchers with prepared data sources, alleviating concerns about data location and the identification of relevant information from the vast pool of available data “outside,” which are often collected by researchers themselves. In the exemplar case described above, the IDM researchers highlighted that during their collaboration with the Washington State DOH, the COVID-19 data shared by the government was of high quality, which significantly expedited their work, enabling them to deliver outputs swiftly [111]. Furthermore, this collaborative model often implies prioritized communication channels, facilitating prompt resolution of data quality issues by both parties.

### Data Management and Sharing

Intentionally built collaborations often smooth data exchange activities by facilitating the creation of a preestablished data sharing framework, which comprises agreements on data management and use, as well as clearly defined obligations and codes of conduct for both parties involved [120]. For example, in response to the external demands for Zika-related research projects, Brazil’s Secretariat of Health took a proactive approach by initiating collaboration protocols with researchers [74]. These data-related agreements clearly regulated the conditions for accessing data and thus laid the foundations for project execution at maximum speed [74]. Besides, such data sharing frameworks function as a common space where collaborators can fine-tune their collective data activities based on project performance.

### Information Systems and Data Interoperability

As government entities and researchers work together around data resources, potential issues, such as inadequate technical infrastructures and inconsistent data standards, may come to light during the early stages [121]. Nonetheless, such a collaboration also presents an opportunity for both parties to acknowledge these problems and proactively work toward resolving them. To illustrate, although not identical to the collaborative model we advocate, the well-known National COVID Cohort Collaborative initiative aimed to overcome interoperability barriers. Ultimately, it managed to build a scalable data analytics infrastructure by uniting US federal agencies, health care providers, and research leaders to harmonize pandemic data across different organizations [122]. Notably, there remains much to investigate regarding the implementation of data standardization within collaborative efforts on a smaller scale, specifically between government entities and researchers.

### Resource Limitations

Government-academic collaborations make dedicated investments, including workforce and funding, in their data projects. This commitment enables the efficient integration of complementary resources from both sectors, facilitating a synergistic approach to data-driven initiatives. In particular, public health and biomedical informatics experts recently stressed the need to build “a public health workforce that is skilled in informatics and data science...to meet 21st century health threats” [21]. Nonetheless, they simultaneously pointed out the challenges in recruiting incoming talent as well as in training this workforce, an ongoing problem that state and local public health departments have historically faced [21]. This further highlights the value of government-academic collaborations, in which public health authorities can borrow well-established expertise from academics in “rapid response data science” [107].

### Multiparty Collaboration and Coordination

Previous ongoing collaborative relationships help to build trust in advance, eliminating the need for a cumbersome initiation period characterized by misaligned organizational priorities and conflicting power dynamics that impede the rapid circulation of data assets. In the case of the Washington State DOH and IDM described above, the IDM researchers achieved favorable outcomes in their collaboration with DOH employees by recognizing the significance of building trust [111]. Based on their accounts, the crucial element that contributed to the success of the collaboration was the ability to align needs and tasks at an early stage of the partnership [111]. In addition, such early coordination efforts may also help data holders preempt the challenges posed by the previously discussed scenario of receiving a sudden surge in requests from data consumers [25].

To summarize, we contend that placing sole emphasis on the exchange of data through technological infrastructures falls short when confronted with the challenges of a public health crisis. Government-academic data collaborations, as essential social infrastructures, encompass not only people, processes, and norms but also rely on trusting relationships within the larger legal and political context. These elements are all integral and indispensable components for the success of the data sharing enterprise. Ultimately, sharing data is not just a technical process—it should be a collaborative endeavor that transcends boundaries. Thus, we should study and implement such collaborations now, before the next public health crisis is on us. Doing so may help to establish greater readiness and more rapid responses in the future. We now outline recommendations that, if implemented, may assist in developing this crucial sociotechnical infrastructure.

## Conclusion and Recommendations

In this viewpoint paper, we investigate the challenges associated with sharing data in public health crises, many of which stem from the long-standing inadequacy in the US public health data infrastructures. In particular, we have witnessed repeated appeals for increased data sharing endeavors spanning various sectors and extending in multiple directions, such as data scientists in the health care industry stressing that “sharing data should not just be a one-way street from the clinician to the researcher” [123]. However, the factors for successful collaborative data sharing across sectors in public health crises—in which government entities share data with academic researchers for effective use—need further attention. Therefore, resulting from a synthesis of extant research and our arguments, we call for more effort to be invested in building data sharing infrastructures capable of bridging and leveraging the respective strengths of government entities and academic researchers. Such infrastructures need to be established within an ecosystem that incorporates not only technologies but also policies, processes, and personnel. This holistic framework is ideally designed to facilitate researchers in seamlessly accessing and employing data aggregated and managed by government entities for their mutual benefit.

The COVID-19 pandemic has taught us a valuable lesson, which surpasses those gained from any previous public health emergencies: that the aforementioned infrastructure for rapid and effective data sharing should be established well in advance of a crisis. Particularly, we argue that government-academic data interactions should not be thought about as only 1-time data sharing. Instead, we recommend that emphasis should be placed on the construction of robust and enduring collaborative infrastructure that not only outlasts a specific public health crisis but also is in place to respond to the next one. Ideally, these data collaborations should not be confined to emergencies or a small number of high-priority threats [51]. After all, data sharing practices both during and between crises affect crisis response efforts, albeit potentially in distinct manners. To be specific, routine data sharing practices in scientific research and the availability of preexisting baseline data before a crisis can play a crucial role in facilitating prompt planning for health relief activities [32,55]. In addition, even before implementing crisis response measures, persistent data partnerships may hold the potential to enhance the detection and early characterization of issues arising during a crisis, facilitated by the accelerated exchange of information between government entities and academics.

As of May 2023, the WHO and the US government declassified COVID-19 as a public health emergency. While most individuals have moved on, for those who have compromised immune systems or are otherwise at greater risk for negative outcomes from the virus, exchanging data to facilitate accurate disease-level reporting remains crucial for evaluating their safety. However, the termination of certain data sharing mandates and data-collection initiatives could hinder government bodies and research institutions from maintaining uninterrupted access to vital disease-related metrics [124]. With the resurgence of COVID-19 hospital admissions since July 2023 in the United States [125] and the possibility of “a new norm of summer surges” [126], it is worth considering whether we want to revert to a just-in-time approach to data sharing practices or if we should be proactive and build just-in-case resilient, long-term data infrastructures for forthcoming public health scenarios. We strongly assert that our choice should be the latter.

## Future Research Agenda

As mentioned, it is critical to begin now to establish more effective government-academic collaborative infrastructures for public health crisis response. To do so, we must develop more systematic research on the facilitating and impeding factors for such data collaborations. In this viewpoint paper, we reviewed existing research literature and summarized data sharing challenges during different crisis scenarios (Table 1). Significantly, we conclude from the literature review that there is a conspicuous scarcity of scholarship addressing the practices and experiences related to disseminating data from government sources to researchers throughout extended or ongoing crisis response situations, including instances of global health crises. In particular, in terms of data-exchange partnerships during public health crises, the public health and biomedical informatics literature often enumerates a wide range of stakeholders [21,127] but generally lacks a specialized focus on the connections between government entities and researchers. On the other hand, literature within the realm of crisis informatics more often addresses the circulation of information and data among the public, frontline responders, and governmental bodies in natural disaster scenarios (eg, earthquakes [88], hurricanes [128], and wildfires [129]).

While government-academic collaborations that allow data exchange did exist at different administrative levels during COVID-19, there is a notable dearth of research studying these relationships. To initiate further discussions, we draw on the data sharing challenges outlined earlier and propose 3 key research questions, to foster more substantive dialogues and shape the future research agenda: (1) What types of government-academic collaborative infrastructures should we be developing? How can these infrastructures be best sustained? (2) Considering the unique characteristics of public health crises, what are the best practices for implementing data sharing and data collaborations? and (3) From the respective views of government entities and researchers, what are the incentives and disincentives that influence their willingness and capacity to engage in developing and sustaining collaborative data infrastructures?

In conclusion, the COVID-19 pandemic has emphasized the imperative for robust and durable government-academic partnerships in public health crises. As we transition beyond the pandemic, it is crucial to develop systematic research on the factors influencing these collaborations. Before the next public health crisis arises, we invite decision makers, researchers, and practitioners across government entities, academia, and various sectors to leverage the collective knowledge and expertise of diverse stakeholders, strengthening existing and building new government-academic data collaborative infrastructures. The time to act is now, and the path to a more resilient future begins with our commitment to addressing these critical challenges.

## Abbreviations

CDC: Centers for Disease Control and Prevention
DOH: department of health
IDM: Institute for Disease Modeling
OGD: open government data
WHO: World Health Organization
